# Sugemalimab plus chemotherapy vs. chemotherapy for metastatic non-small-cell lung cancer: A cost-effectiveness analysis

**DOI:** 10.3389/fpubh.2023.1054405

**Published:** 2023-02-27

**Authors:** Xueyan Liang, Xiaoyu Chen, Huijuan Li, Xiaoxia Liu, Yan Li

**Affiliations:** Department of Pharmacy, Guangxi Academy of Medical Sciences and the People's Hospital of Guangxi Zhuang Autonomous Region, Nanning, Guangxi, China

**Keywords:** sugemalimab, partitioned survival model, non-small cell lung cancers, chemotherapy, cost-effectiveness

## Abstract

**Background:**

Sugemalimab is a newly developed inhibitor of programmed death ligand 1 (PD-L1). As a first-line treatment for metastatic non-small-cell lung cancer (NSCLC), sugemalimab plus chemotherapy (Sugema-Chemo) has been proven effective. Still, its cost-effectiveness has not yet been determined. The objective of this study was to assess the cost-effectiveness of Sugema-Chemo from a health care perspective in China.

**Methods:**

A partitioned survival model was used. According to the GEMSTONE-302 trial, the clinical characteristics and outcomes of the patients were obtained. The outcomes were costs, quality-adjusted life-years (QALYs), incremental cost-effectiveness ratio (ICER), incremental net health benefits (INHB) and incremental net monetary benefits (INMB). The robustness of the model was further evaluated, as well as subgroup analyses. When the ICER was lower than the willingness to pay (WTP) threshold ($38,017/QALY or $86,376/QALY, defined as three times the per capita gross domestic product value of the general region and Beijing), the cost-effectiveness of Sugema-Chemo was assumed for general regions or Beijing.

**Results:**

Compared with chemotherapy alone, Sugema-Chemo resulted in an incremental gain of 0.82 QALYs, an incremental gain of 1.26 life-years, as well as an average increase cost of $72,472. The ICER was $88,744/QALY. Model outcomes were susceptible to average body weight and cost of sugemalimab. Sugema-Chemo was cost-effective at a WTP threshold of 86,376/QALY if the average body weight was <62.44 kg or if the price of sugemalimab was <$2.996/mg. As well, Sugema-Chemo was also cost-effective when the cost of sugemalimab was <$1.839/mg for a WTP threshold of $38,017/QALY. Sugema-Chemo had a probability of > 50% being considered cost-effective in most subgroups at the $86,376/QALY threshold. However, Sugema-Chemo did not achieve cost-effectiveness (0%) in any of the subgroups when WTP was set at $38,017/QALY.

**Conclusion:**

Sugema-Chemo might not be cost-effective in patients with metastatic NSCLC in China. In deciding between Sugema-Chemo and chemotherapy alone, it is essential to consider both the body weight of patients and the price of sugemalimab. A price reduction of sugemalimab under the National Healthcare Security Administration may be an effective measure to improve the cost-effectiveness of the drug.

## Introduction

Lung cancer is one of the most common types of cancer, and it is the leading cause of cancer death worldwide (1). Nearly 85% of lung cancers are non-small cell lung cancers (NSCLC) (2). Squamous and non-squamous lung cancers account for 25–30% and 70–75% of all cases of NSCLC, respectively (1, 3). NSCLC is generally diagnosed at an advanced stage and has a poor prognosis for most patients (4). Platinum-based chemotherapy has traditionally been recommended as first-line treatment for patients with advanced NSCLC without targetable genetic alterations (5). However, there was a limited survival benefit associated with these interventions and the median overall survival (OS) was <1 year for these interventions (6). There is an urgent need for novel interventions to improve survival rates in advanced NSCLC, considering its prevalence and poor outcomes (4, 6).

In the past few years, immune checkpoint inhibitors (ICIs) have demonstrated promising benefits and a favorable safety profile, so they are rapidly incorporated into the standard treatment for advanced NSCLC (7). Sugemalimab is a humanized monoclonal antibody that inhibits programmed death ligands (8). As first-line therapy for patients with metastatic NSCLC, sugemalimab plus chemotherapy (Sugema-Chemo) significantly prolonged progression-free survival (PFS) in the GEMSTONE-302 randomized phase 3 trial compared to chemotherapy alone, as well as increasing response rates (8). Accordingly, Sugema-Chemo is a promising first-line treatment option for metastatic NSCLC. Sugemalimab, in combination with pemetrexed and carboplatin, has been approved in China for treating non-squamous NSCLC, and for patients with squamous NSCLC (8, 9).

Despite this, due to the relatively high cost of the combination therapy (Sugema-Chemo), it is urgent to perform a cost-effectiveness analysis of Sugema-Chemo compared to chemotherapy for treating NSCLC. Therefore, the purpose of the present study was to investigate the cost-effectiveness of Sugema-Chemo as first-line therapy for metastatic NSCLC in comparison with chemotherapy alone from a health care perspective in China.

## Materials and methods

### Patients and intervention

This study was conducted following the Consolidated Health Economic Evaluation Reporting Standards (CHEERS) (10). Patients with metastatic NSCLC enrolled in the GEMSTONE-302 study were the target population (8). GEMSTONE-302 is designed to enroll patients with metastatic non-squamous or squamous NSCLC who are at least 18 years of age without known EGFR sensitizing mutations, ALK, ROS1, or RET fusions and who have not received any prior systemic therapy for metastatic disease. The study enrolled individuals who received sugemalimab 1,200 mg once every 3 weeks or placebo, in combination with carboplatin and paclitaxel for patients with squamous NSCLC, or combination with carboplatin and pemetrexed for patients with non-squamous NSCLC. The maintenance treatment for squamous NSCLC consisted of sugemalimab or placebo, and the maintenance treatment for non-squamous NSCLC consisted of sugemalimab or placebo.

### Model structure

We conducted an economic evaluation and used a partitioned survival model that considers three mutually exclusive health states: progression-free survival (PFS), progressive disease (PD), and death (11). Both treatment arms had a 15-year time horizon, and more than 98% of patients died during this period. The cycle length was 1-week. Based on clinical results from the GEMSTONE-302 trial, the proportions of patients with OS and PFS were established in the model (8). A portion of the OS curve was evaluated for the proportion of patients still alive; the portion of the PFS curve was evaluated for the proportion of patients living with PFS, and the difference between OS and PFS curves was evaluated for the proportion of patients living with PD. This study did not require or obtain an institutional review board review or informed consent because data were obtained from the literature and open databases.

### Clinical data inputs

According to Guyot et al.s' algorithm, the OS and PFS of the GEMSTONE-302 trial were extrapolated beyond the follow-up period of the trial using the OS and PFS data obtained from the trial (12). To obtain the individual patient data points, the Kaplan-Meier (K-M) survival curves for OS and PFS were calculated using GetData Graph Digitizer version 2.26 (13). After calculating these data points, we fitted them with parametric survival functions: exponential, Weibull, gamma, lognormal, Gompertz, log-logistic, and generalized gamma. Afterward, the best-fit parametric models for the reconstructed K-M survival curves were selected based on Akaike Information Criterion (AIC) and Bayesian Information Criterion (BIC). Sugema-Chemo and chemotherapy survival functions and parameterized models are shown in Table 1, while goodness-of-fit results are shown in Supplementary Table 1. Specifically, Log-logistic was selected to fit the PFS K-M curves of Sugema-Chemo or chemotherapy, and the OS K-M curves of Sugema-Chemo. Lognormal was selected to fit the OS K-M curves of chemotherapy alone (Supplementary Figure 1). The key clinical input data are listed in Table 1.

**Table 1:** Key model inputs.

**Parameter**	**Value (95% CI)**	**Distribution**	**References**
**Clinical input**			
Survival model for sugemalimab plus chemotherapy			
Log-logistic model for OS^a^	γ = 1.3113	ND	(8)
	λ = 0.0253		
Log-logistic model for PFS^a^	γ = 1.6344	ND	(8)
	λ = 0.0094		
Survival model for chemotherapy			
Lognormal model for OS^a^	μ = 4.2489	ND	(8)
	σ = 1.0432		
Log-logistic model for PFS^a^	γ = 2.1146	ND	(8)
	λ = 0.0446		
**Cost input**			
Drug costs per 1 mg			
Sugemalimab	3.05 (2.44–3.66)	Gamma	Local database
Carboplatin	0.12 (0.08–0.16)	Gamma	Local database
Pemetrexed	1.17 (0.22–3.19)	Gamma	Local database
Paclitaxel	0.80 (0.32–1.16)	Gamma	Local database
Nivolumab	15.44 (13.79–17.09)	Gamma	Local database
Docetaxel	1.61 (0.73–2.25)	Gamma	Local database
Second-line treatment in sugemalimab plus chemotherapy arm per cycle	789 (631–947)	Gamma	(8); Local database
Second-line treatment in chemotherapy arm per cycle	1,512 (1,210–1,814)	Gamma	(8); Local database
Cost of terminal care per patient^b^	16,441.83 (12,331.37–20,552.29)	Gamma	(14)
Disease costs per cycle			
Patients with PFS^c^	464.85 (348.64–581.06)	Gamma	(14)
Patients with PD^c^	1,075.49 (806.62–1,344.36)	Gamma	(14)
Cost of managing AEs (grade ≥ 3)			
Sugemalimab plus chemotherapy	3,063 (2,450.4–3,675.6)	Gamma	(15–17)
Chemotherapy	2,984 (2,387.2–3,580.8)	Gamma	(15–17)
Supportive care per cycle^d^	72 (58–86)	Gamma	(18)
Cost of drug administration per unit	19.11 (15.288–22.932)	Gamma	(19)
**Health utilities**			
Disease status utility per year			
Utility of PFS	0.804 (0.64–0.96)	Beta	(20)
Utility of PD	0.321 (0.26–0.39)	Beta	(20)
Death	0	NA	
Disutility due to AEs			
Sugemalimab plus chemotherapy	0.159 (0.119–0.199)	Beta	(20–22)
Chemotherapy	0.147 (0.110–0.184)	Beta	(20–22)
Body surface area, m^2^	1.8 (1.44–2.16)	Normal	(23, 24)
Body weight, kg	65 (50–90)	Normal	(23, 24)
Creatinine clearance rate, ml/min/1.73 m^2^	90 (80–12)	Normal	(23)

ND, not determined; OS, overall survival; PD, progressed disease; PFS, progression-free survival; AEs, adverse events.

^a^Only expected values are presented for these survival model parameters.

^b^Overall total cost per patient regardless of treatment duration.

^c^It was assumed that these costs would continue until the health state transitioned.

^d^Physician visits, laboratory tests, and examinations were all included in the routine follow-up cost.

### Cost inputs

Several direct medical costs have been evaluated, including those associated with obtaining drugs, the cost of supportive care, the cost of terminal care, and the cost of adverse events (AEs) (Table 1). We obtained the prices in Chinese Yuan and translated them into US dollars using the exchange rate of 2021 (1 US dollar = 6.37 Chinese Yuan) (25). Based on the standard fee database, the drug costs were determined. We assume that the average body surface area (BSA), weight, and creatinine clearance rate (CCR) were 1.80 m^2^, 65 kg, and 90 ml/min/1.73 m^2^. Those assumption were used to calculate the median dosage of chemotherapy and sugemalimab (23, 24). In addition, the costs for managing Grade ≥ 3 AEs were calculated by multiplying the rates contained in the randomized controlled trial, and the management costs were derived from the literature (Supplementary Table 2) (15–17). Approximately 141 patients (84%) with radiographic progression in the Sugema-Chemo group and 99 patients (86%) with radiographic progression in the chemotherapy group would receive subsequent treatment in the GEMSTONE-302 trial. Based on the lack of detailed information collected in the preliminary trial, we adopted subsequent treatment strategies recommended by the National Comprehensive Cancer Network (NCCN) (26) and the Chinese Society of Clinical Oncology (CSCO) guidelines (27, 28) (Table 1). According to NCCN and CSCO guidelines, nivolumab or docetaxel were used as the subsequent treatment strategies for NSCLC. Based on the published literature, we determined costs related to disease (14), subsequent supportive care (18), terminal care (14), and drug administration (19) during the study.

### Utility inputs

There was a range of health utility scores between 0 (death) and 1 (perfect health). Because health utilities for PFS and PD were not included in GEMSTONE-302, we have adopted health utilities from the clinical literature (20). In relation to NSCLC, the utilities of PFS and PD were 0.804 and 0.321, respectively (20). Additionally, disutility values associated with AEs were determined based on the literature (Supplementary Table 2) (20–22).

### Base-case analysis

We calculated an incremental cost-effectiveness ratio (ICER), which is the incremental cost per quality-adjusted life year (QALY) gained. We calculated ICERs based on two willingness to pay (WTP) thresholds in consideration of the imbalance in economic development among Chinese socioeconomic regions: three times the per capita gross domestic product (GDP) value of China in 2021 ($38,017/QALY) for general regions, and three times Beijing's per capita GDP value in 2021 ($86,376/QALY) for affluent regions (29). Costs and utility outcomes were discounted at a rate of 5% annually (30). Moreover, we calculated the incremental net health benefit (INHB) as well as the incremental monetary benefit (INMB) (31).

### Sensitivity analysis

We conducted a one-way sensitivity analysis in this study in order to identify significantly sensitive variables and evaluate the robustness of the results. Several variables, such as costs and utilities, were subjected to one-way sensitivity analyses, and the uncertainty of each variable was calculated using 95% confidence intervals reported in the literature or estimated by assuming a 20% variation from the fundamental variables (Table 1). Monte Carlo simulations were used to conduct a probabilistic sensitivity analysis with 10,000 iterations. Three distributions were assigned to the parameters in the model: gamma, log-normal, and beta distributions. Gamma distributions were assigned to the cost parameters, log-normal distributions to the hazard ratios (HRs), and beta distributions to the proportions and probabilities. We then generated a cost-effectiveness acceptability curve to illustrate the possibility that Sugema-Chemo or chemotherapy could be considered a cost-effective option at different WTP levels in terms of per QALYs gained.

### Subgroup analysis

We performed a subgroup analysis in order to determine whether different characteristics of patients contribute to the uncertainty of outcomes for each of the subgroups obtained from the GEMSTONE-302 by various HR for PFS (8). In this study, statistical analyses were carried out using R (version 4.0.5, 2021, R Foundation for Statistical Computing) with hesim and heemod packages.

## Results

### Base-case analysis

The Sugema-Chemo combination resulted in 0.82 QALYs gain and 1.26 overall life-years gain for patients with NSCLC in the base-case analysis, at additional costs of $72,472 compared to chemotherapy alone, which corresponded to an ICER of $88,744/QALY. In addition, at the $38,017/QALY WTP threshold, Sugema-Chemo had an INHB and an INMB of −1.09 QALYs and –$41,298, respectively, compared to chemotherapy alone. Furthermore, Sugema-Chemo had an INHB of −0.02 QALYs and an INMB of -$1,644 when the WTP threshold was set at $86,376/QALY (Table 2).

**Table 2:** Summary of cost and outcome results in the base-case analysis.

**Factor**	**Sugemalimab plus chemotherapy**	**Chemotherapy**	**Incremental change^a^**
**Cost, $**			
Drug	183,444	115,509	67,935
Non-drug^b^	13,435	8,898	4,537
AEs management	637	411	226
Best supportive care	11,759	7,798	3,961
Overall	196,879	124,407	72,472
**Life-years**			
Progression-free	1.8	0.63	1.17
Overall	3.49	2.23	1.26
**QALYs**	1.76	0.94	0.82
**ICERs, $**			
Per life-year	NA	NA	57,706
Per QALY	NA	NA	88,744
INHB, QALY, at threshold 38,017^a^	NA	NA	−1.09
INMB, $, at threshold 38,017^a^	NA	NA	−41,298
INHB, QALY, at WTP threshold 86,376^a^	NA	NA	−0.02
INMB, $, at WTP threshold 86,376^a^	NA	NA	−1,644

AEs, adverse events; ICERs, incremental cost-effectiveness ratios; INHB, incremental net health benefit; INMB, incremental net monetary benefit; NA, not applicable; QALY, quality-adjusted life-year; WTP, willingness-to-pay.

^a^Sugemalimab plus chemotherapy versus chemotherapy.

^b^Non-drug cost includes the costs of AEs management, subsequent best supportive care per patient, follow-up care covering physician monitors, drug administration, and terminal care.

### Sensitivity analysis

**Figure 1:**
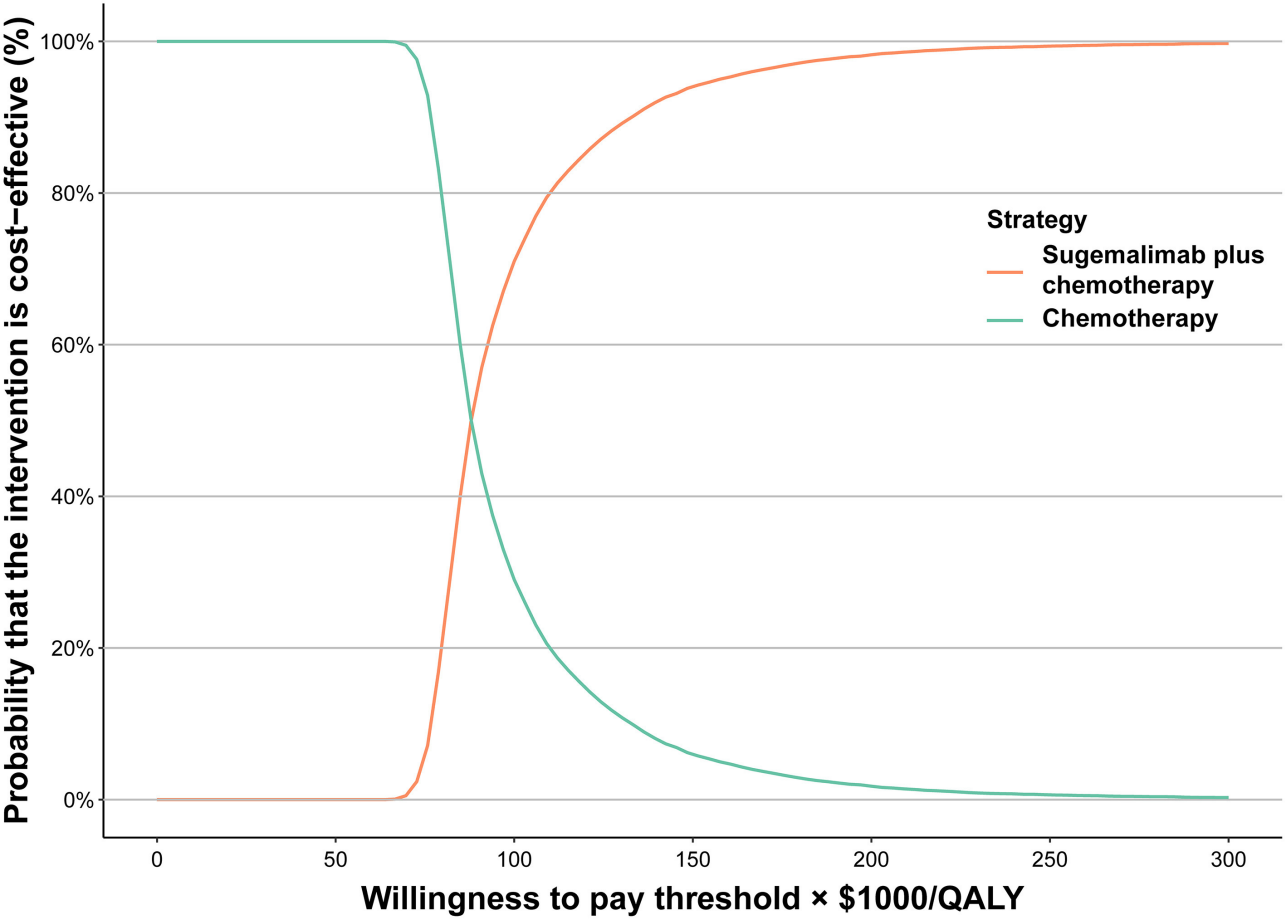
Acceptability curves of cost-effectiveness. QALY, quality-adjusted life-year.

According to the one-way sensitivity analysis, the model outcome was largely driven by the average body weight, the utility for PFS, the cost of sugemalimab, and HR for OS (Sugema-Chemo vs. chemotherapy). There was a marginal relationship between the remaining parameters and outcomes (Supplementary Figure 2). We also examined the relationship between these key variables and the ICER between Sugema-Chemo and chemotherapy alone. Sugema-Chemo was cost-effective when the average body weight was lower than 62.44 kg, the utility of PFS exceeded 0.826, Sugemalimab was purchased at <$2.996/mg, or the HR for OS exceeded 0.711 for a WTP threshold of 86,376/QALY. Otherwise, chemotherapy was preferred. The results also demonstrated that Sugema-Chemo may be cost-effective at the WTP threshold of $38,017/QALY when sugemalimab costs <$1.839/mg; otherwise, chemotherapy was preferred (Supplementary Figure 3). Based on the cost-effectiveness acceptability curve, Sugema-Chemo has a 60% probability to be considered cost-effective, at the WTP threshold of $86,376/QALY. Nevertheless, Sugema-Chemo did not have a chance of being considered cost-effective if the WTP threshold was $38,017/QALY (Figure 1).

### Subgroup analysis

**Figure 2:**
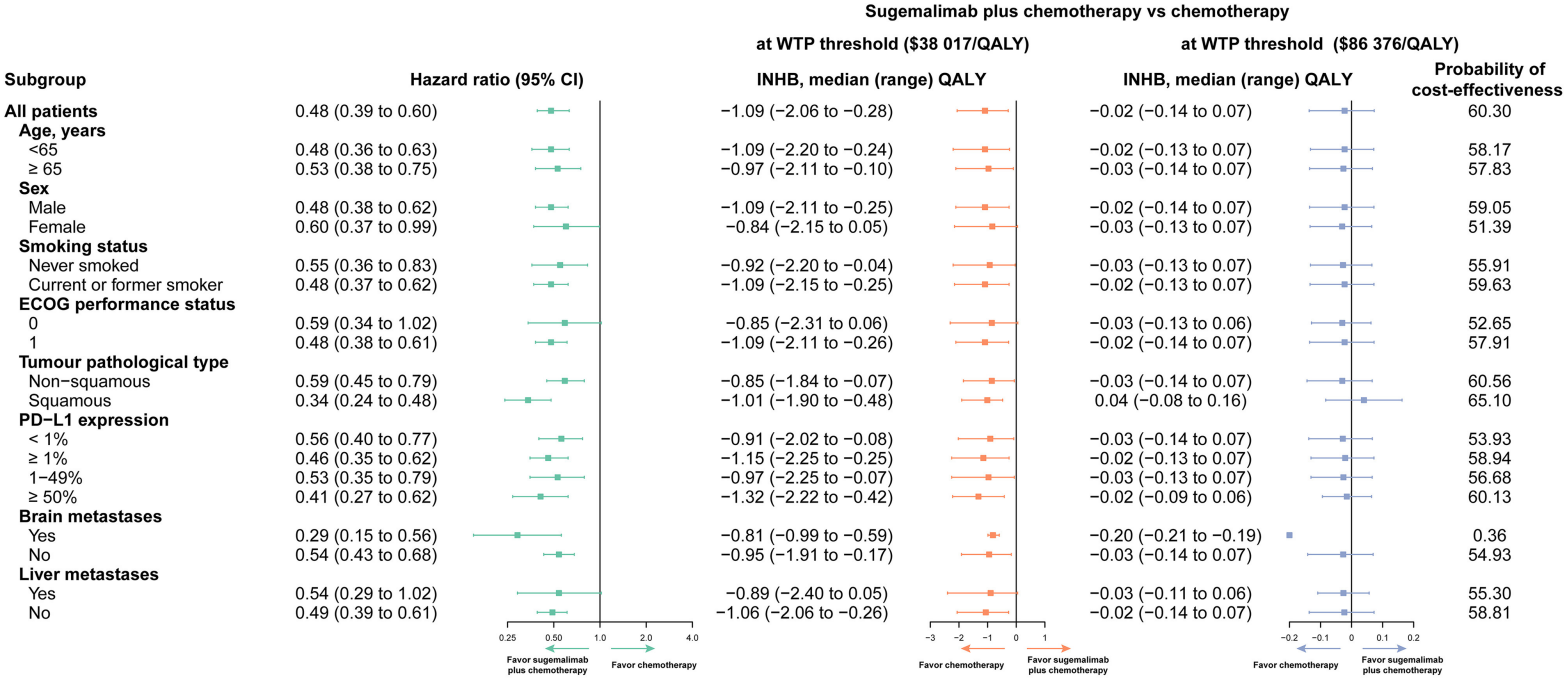
Subgroup analyses obtained by varying the hazard ratios (HRs) for progression-free survival (PFS). INHB, incremental net health benefit; WTP, willingness to pay; QALY, quality-adjusted life-year; PD-L1, programmed cell death ligand 1.

Variations in the HR for PFS were used for the subgroup analysis. Compared to chemotherapy, the Sugema-Chemo group demonstrated a more significant reduction in death risk. Further, Sugema-Chemo was not significantly superior to chemotherapy in improving PFS in patients with ECOG performance status 0 and with liver metastases. Most subgroups, except for the patients with brain metastases, were likely to consider Sugema-Chemo cost-effective at the WTP threshold of $86,376/QALY. In all subgroups evaluated, Sugema-Chemo was not cost-effective (0%) at a WTP threshold of $38,017/QALY (Figure 2).

## Discussion

This study is the first to compare the cost-effectiveness of Sugema-Chemo to chemotherapy alone in patients with metastatic NSCLC in China. In the GEMSTONE-302 trial, Sugema-Chemo statistically extended PFS in patients with metastatic NSCLC compared with chemotherapy alone. Nevertheless, due to the high price of sugemalimab, physicians and patients are uncertain about which is more beneficial. It is urgent to conduct a cost-effectiveness analysis to evaluate the efficacy and cost of Sugema-Chemo.

With a large population and rapid development, China is one of the most populous countries in the world. Therefore, the imbalance between economic development at the provincial level is therefore an objective fact. Taking into consideration regional economic disequilibrium, the WTP threshold for general regions and affluent regions of China was set at $38,017/QALY and $86,376/QALY, respectively. The combined treatment of Sugema-Chemo may not be a cost-effective alternative to chemotherapy alone, as the ICER of $88,744/QALY is higher than the WTP threshold of $38,017/QALY. There was, however, a 60% probability that this ICER would be considered a cost-effective option if it approached the WTP threshold of $86,376/QALY. One-way sensitivity analysis and probabilistic sensitivity analysis indicate that the results of this model are robust. An analysis of the sensitivity of the model indicated that it was susceptible to the average body weight, as well as the utility for PFS, cost of sugemalimab, and HR for OS. With a WTP threshold of 86,376/QALY, Sugema-Chemo was cost-effective if the average body weight was <62.44 kg, or the price was <$2.996/mg. In addition, for a WTP threshold of $38,017/QALY, Sugema-Chemo is cost-effective if the price of sugemalimab is <$1.839/mg; otherwise, chemotherapy is the preferred option.

As far as we know, since the official establishment of the National Healthcare Security Administration (NHSA) in May 2018, there have been several rounds of negotiations with pharmaceutical companies on the price of cancer drugs, aiming to relieve the medical burden of cancer patients through national strategic procurement (32). The NHSA in China has made a great effort to negotiate drug prices with pharmaceutical companies, with the result that the prices of many anticancer drugs have been reduced by 30–70% (32). Considering the circumstances, it is unlikely that a rise in the price of sugemalimab. On the contrary, if negotiations for sugemalimab are conducted, the cost of sugemalimab is highly likely to decrease. As a result, our findings indicate that Sugema-Chemo can provide adequate first-line treatment for patients with advanced NSCLC within an appropriate price range in a cost-effective manner. The NHSA negotiation will be the most effective approach for optimizing the allocation of medical resources in China for an extended period, providing patients with better health services at low costs (33).

This study has several advantages worth highlighting. First, to our knowledge, this is the first study to examine the cost-effectiveness of Sugema-Chemo combination therapy in treating metastatic NSCLC using a partitioned survival model based on the latest published GEMSTONE-302 trial. Second, at current prices, Sugema-Chemo combination is unlikely to be an attractive cost-effective option over chemotherapy alone. However, clinical trial results indicate that it increases OS and PFS in patients with metastatic NSCLC. Third, the patient population evaluated in the trial was Chinese, which means that race did not influence on the results. Additionally, the partitioned survival model did not require assumptions for the transition of patients between health states but made it possible to directly partition patients into different health states based on the K-M curves. Last, the present study analyzed the economic outcomes of 18 subgroups evaluated by the GEMSTONE-302 trial. Physicians, patients, and policymakers may benefit from the economic results of the subgroups.

There have some limitations in the analysis. The parameter distributions fitted to the K-M curves were assumed to be effective outcomes that exceeded the follow-up period of the GEMSTONE-302 trial, leading to uncertainty in the model outputs. A sensitivity analysis revealed that this finding is generally robust, indicating that this limitation may not be a significant factor. It is also important to note that there is inherent uncertainty when extrapolating PFS and OS over the longer term. Second, the clinical data included in the model have been derived from the results of GEMSTONE-302 trial. Therefore, any biases within the trial may have affected the results of the trial in terms of cost and effectiveness. Accordingly, the characteristics of patients with NSCLC included in the GEMSTONE-302 study were generally strict. Additionally, clinical trial participants tend to adhere to their treatment regimens more closely than patients in real-world practice. Moreover, with a median follow-up of 8.6 months (IQR 6.1–11.4), the preplanned interim analysis of the GEMSTONE-302 trial and the prediction of cost-effectiveness could potentially be altered if more follow-up data are available. Third, the values of utility and disutility were derived from published literature, some of the data were not obtained from Chinese populations, and bias was not distinguished due to of different treatment strategies (20). Based on the data, NSCLC utility values in China were higher than those in other countries.

## Conclusion

Based on the health care perspective in China, this study indicates that Sugema-Chemo may not be cost-effective as the first-line treatment for metastatic NSCLC patients at a WTP threshold of $38,017 or $86,376/QALY and under current drug pricing. Sengemalimab may be economically advantageous if its price is reduced substantially. If individual treatments are tailored based on the factors contributing to the economic outcome, the economicoutcomes may be improved. In treating patients with metastatic NSCLC, the results of this study may assist clinicians in choosing appropriate treatments.

## Data availability statement

The original contributions presented in the study are included in the article/Supplementary material, further inquiries can be directed to the corresponding author.

## Author contributions

Gathering and analyzing all data: YL. Concept and design: XLia, YL, and XC. Drafting and statistical analysis: YL and XLia. Funding: XC. Technical and material support and supervision: HL and XLiu. Data interpretation and critical revision of the manuscript: all authors. All authors contributed to the article and approved the submitted version.
