# Supplementary material for: Sugemalimab plus chemotherapy vs. chemotherapy for metastatic non-small-cell lung cancer: A cost-effectiveness analysis
Source: Front Public Health. 2023 Feb 27;11:1054405. doi: 10.3389/fpubh.2023.1054405 (PMC10008846; doi:10.3389/fpubh.2023.1054405)
Supplement: Supplementary file 1 [file Data_Sheet_1.PDF]

Supplementary Content

Supplementary Figure 1. Model Fitting Analysis

Supplementary Figure 2. Tornado Diagram of One-Way Sensitivity Analysis

Supplementary Figure 3. Impacts of Key Factors on ICER

Supplementary Table 1. Evaluated Parameters and Values of AIC and BIC

Supplementary Table 2. Probability and Costs Associated with Adverse Events (Grade ≥3)

Supplementary Figure 1. Model Fitting Analysis

To obtain the best model fit, the following investigations were carried out using sugemalimab plus chemotherapy or chemotherapy as the model fit baseline, respectively. Based on AIC and BIC (Supplementary Table 1), log-logistic was used to fit the OS K-M of sugemalimab plus chemotherapy. Log-logistic was used to fit the PFS K-M of sugemalimab plus chemotherapy arm, as well as chemotherapy arm. Lognormal was used to fit the OS K-M of chemotherapy arm.

(A) Model-fitted versus original K-M curves for sugemalimab plus chemotherapy.

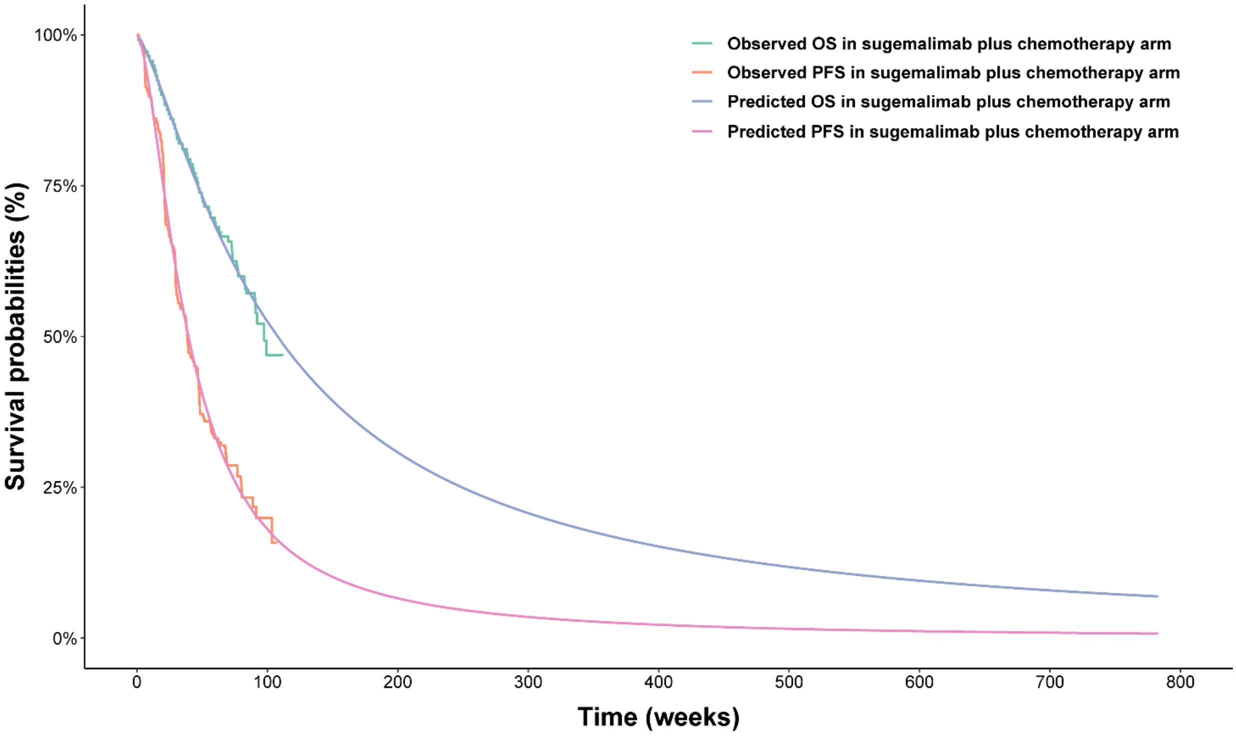

(B) Model-fitted versus original K-M curves for chemotherapy.

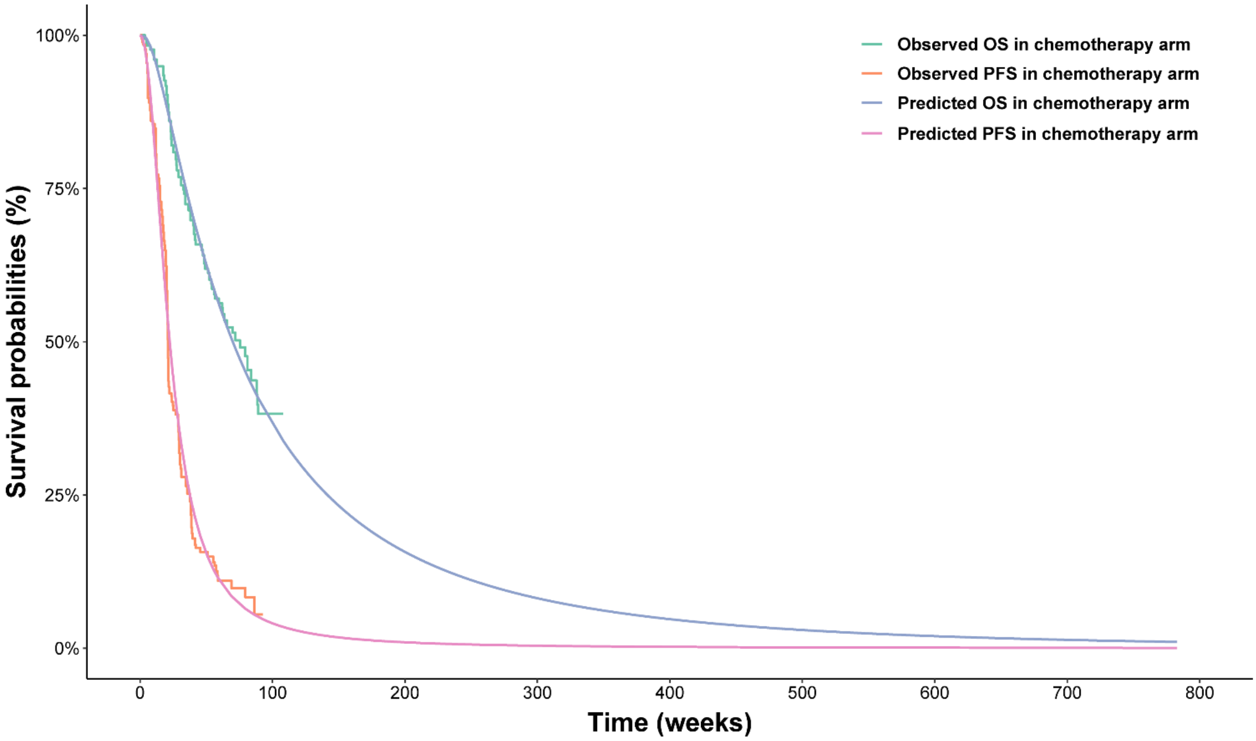

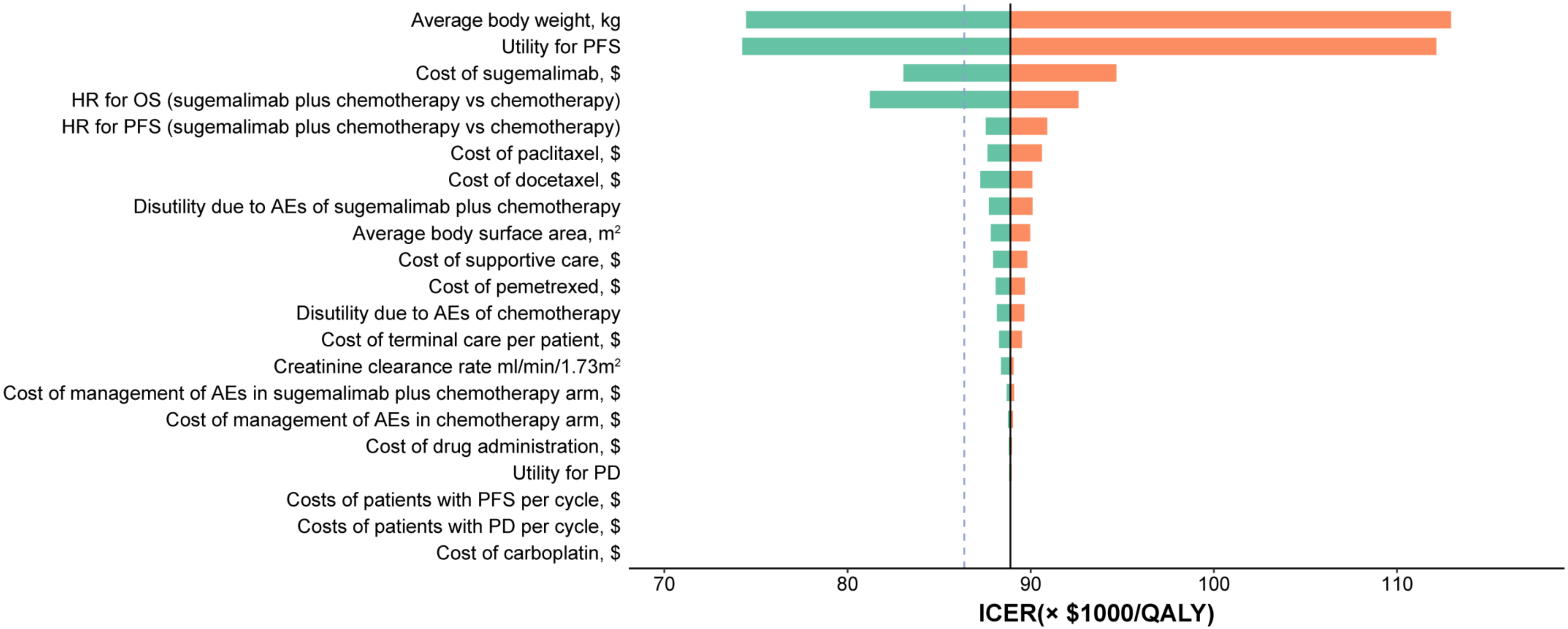

Supplementary Figure 2. Tornado Diagram of One-Way Sensitivity Analyses. OS, overall survival; HR, hazard ratio; PD, progressed disease; PFS, progression-free survival; AEs, adverse events.

Supplementary Figure 3. Impacts of Key Factors on ICER

The diagrams show the impacts of key factors on the incremental cost-effectiveness ratio for the treatment of metastatic NSCLC. (A) represents the impacts of body weight; (B) represents the impacts of utility for PFS; (C) represents the impacts of the cost of sugemalimab; (D) represents the impacts of HR for OS. ICER, incremental cost-effectiveness ratio; QALY: quality-adjusted life-year.

(A) represents the impacts of body weight

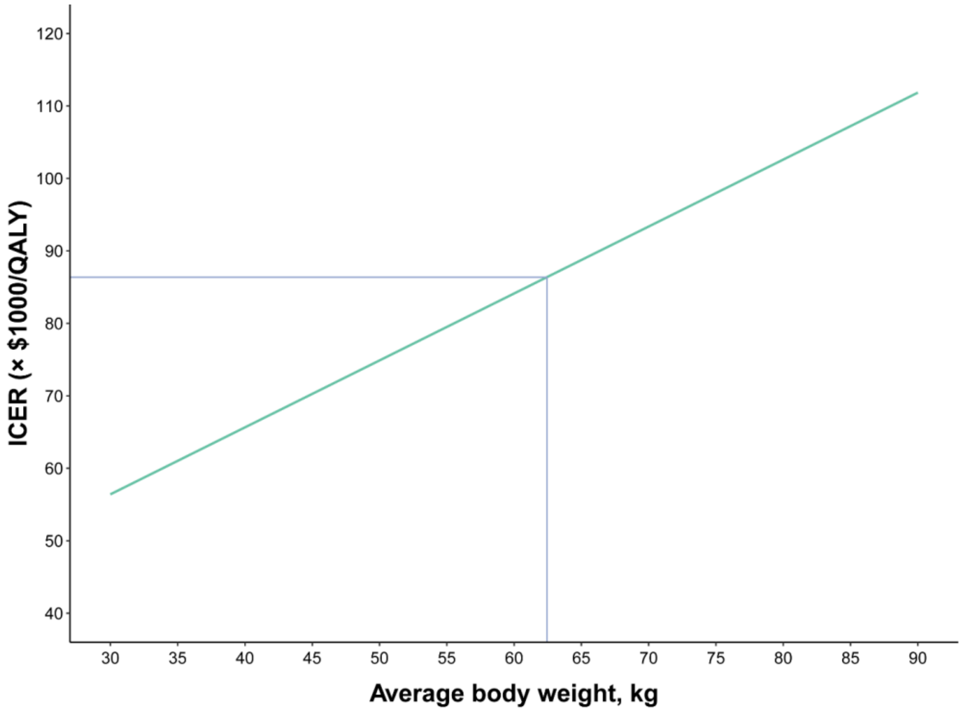

(B) represents the impacts of utility for PFS

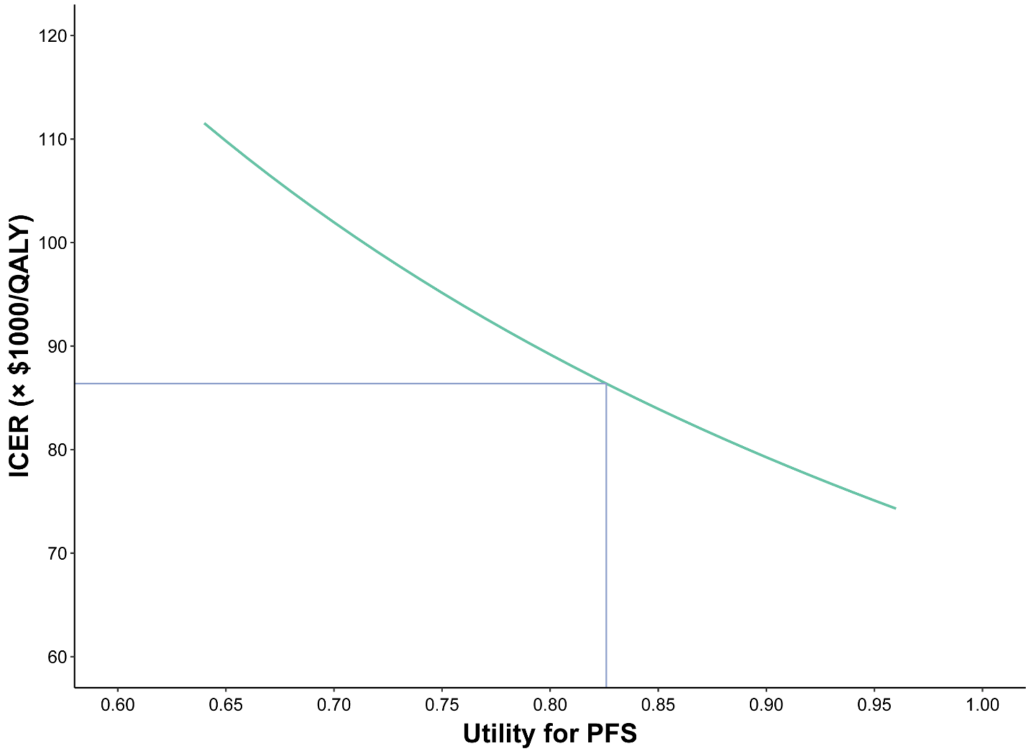

(C) represents the impacts of the cost of sugemalimab

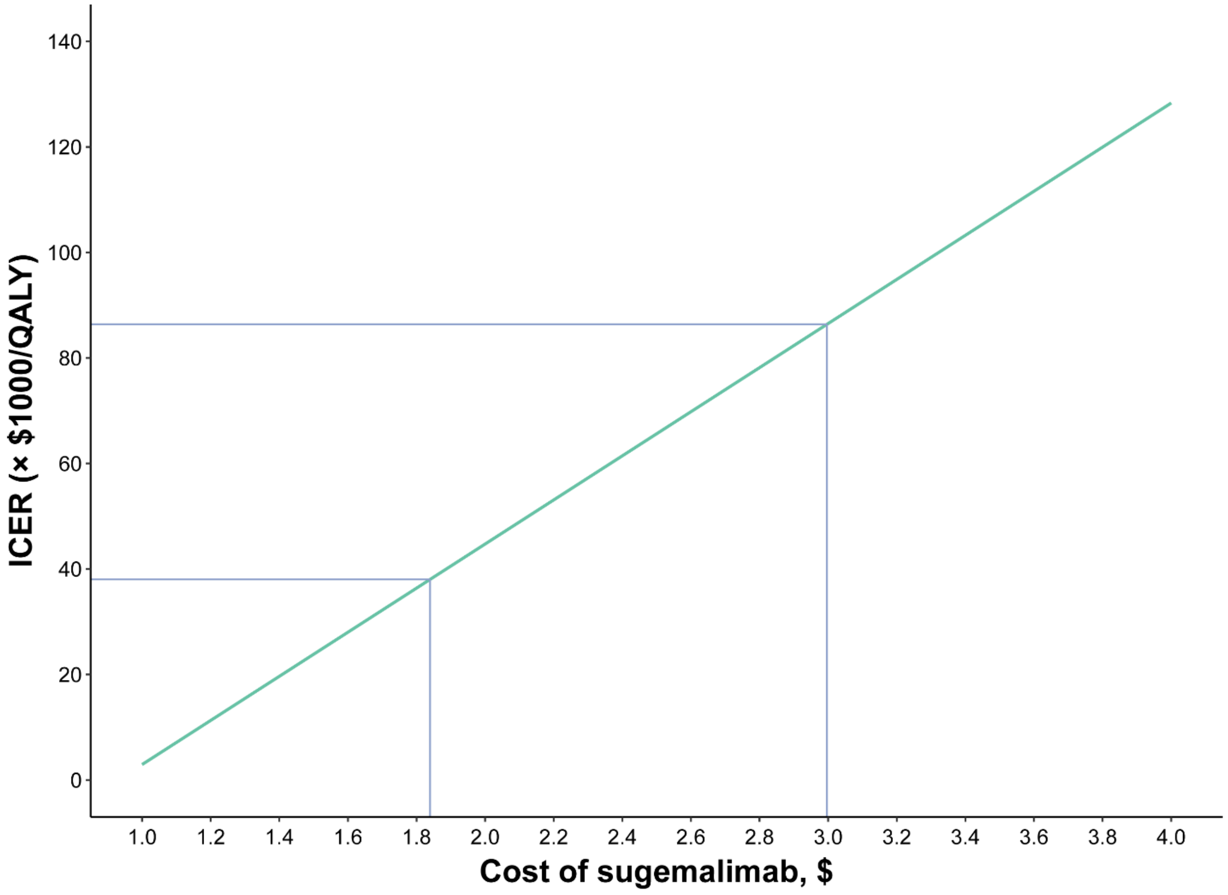

(D) represents the impacts of HR for OS

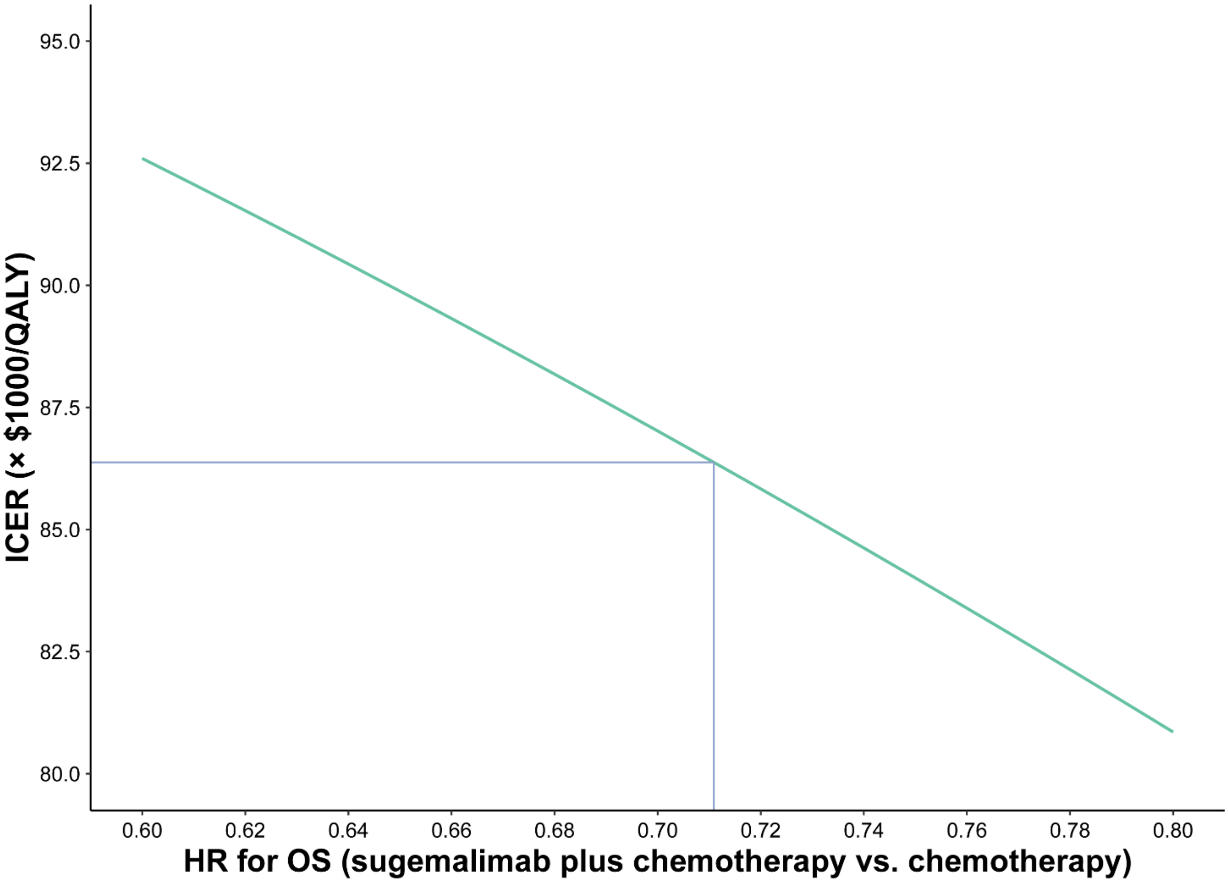

Supplementary Table 1. Evaluated Parameters and Values of AIC and BIC

Strategies	Distributions	Parameters	est	se	L95%	U95%	AIC	BIC
**Results of OS**								
**Sugema-Chemo**	Exponential	rate	0.00637976	0.0005706	0.0053539	0.0076022	1515.656	1519.424
	WeibullPH	shape	1.16385977	0.0951193	0.9915946	1.3660518	1515.711	1523.248
		scale	0.00317378	0.001321	0.0014037	0.0071758		
	Gamma	shape	1.20855079	0.1261191	0.9850023	1.4828341	1514.489	1522.026
		rate	0.00869256	0.0015906	0.0060728	0.0124425		
	Lognormal	meanlog	4.76503	0.116341	4.53701	4.99306	1521.876	1529.412
		sdlog	1.45898	0.102276	1.27168	1.67386		
	Gompertz	shape	0.0058545	0.0034592	-0.000925	0.0126344	1514.847	1522.383
		rate	0.0050713	0.0008524	0.0036479	0.00705		
	Log-logistic	shape	1.31129	0.10433	1.12195	1.53257	**1514.403**	**1521.94**
		scale	107.80411	10.38969	89.24835	130.21783		
	Generalized gamma	mu	4.952002	0.110468	4.7354889	5.16852	1516.384	1527.689
		sigma	0.813753	0.302715	0.3925062	1.68709		
		Q	1.08969	0.599515	-0.085338	2.26472		
**Chemo**	Exponential	rate	0.00941572	0.0010527	0.0075629	0.0117225	908.46	911.529
	WeibullPH	shape	1.381073	0.1339174	1.1420335	1.670146	900.731	906.869
		scale	0.001915	0.0011022	0.0006198	0.0059166		
	Gamma	shape	1.6277867	0.2248333	1.2417317	2.133867	899.227	905.365
		rate	0.0185603	0.0037173	0.0125344	0.027483		
	Lognormal	meanlog	4.24885	0.1017058	4.04951	4.44819	**896.625**	**902.762**
		sdlog	1.04317	0.0891851	0.882231	1.23347		
	Gompertz	shape	0.00933233	0.0043615	0.000784	0.0178806	906.02	912.158
		rate	0.00664996	0.0013835	0.0044232	0.0099978		
	Log-logistic	shape	1.67223	0.157692	1.39005	2.01171	898.113	904.251
		scale	69.24402	6.529695	57.55912	83.30103		
	Generalized gamma	mu	4.2745498	0.175017	3.931522	4.617577	898.593	907.8
		sigma	1.021925	0.150822	0.765232	1.364724		
		Q	0.0770379	0.434813	-0.77518	0.929255		

Results of PFS								
**Sugema-Chemo**	Exponential	rate	0.0170203	0.0011372	0.0149312	0.0194017	2274.86	2278.629
	WeibullPH	shape	1.22097361	0.0687687	1.0933625	1.3634787	2265.344	2272.881
		scale	0.00708212	0.0020047	0.0040665	0.0123342		
	Gamma	shape	1.405713	0.1182631	1.1920229	1.6577094	2261.721	2269.258
		rate	0.025793	0.0029511	0.0206116	0.0322768		
	Lognormal	meanlog	3.67099	0.0648732	3.543845	3.79814	2257.691	2265.228
		sdlog	1.08047	0.0539248	0.979781	1.1915		
	Gompertz	shape	0.00388275	0.0028979	-0.001797	0.0095624	2275.108	2282.644
		rate	0.01514544	0.0016982	0.0121574	0.0188679		
	Log-logistic	shape	1.63443	0.0922724	1.46322	1.82566	**2253.514**	**2261.051**
		scale	39.54008	2.4133577	35.08196	44.56473		
	Generalized gamma	mu	3.801033	0.1035907	3.597999	4.004067	2257.45	2268.755
		sigma	1.003374	0.0746356	0.867254	1.160858		
		Q	0.315985	0.2093855	-0.094403	0.726373		
**Chemo**	Exponential	rate	0.031708	0.0027392	0.0267692	0.0375579	1194.918	1197.987
	WeibullPH	shape	1.32083737	0.0872388	1.1604572	1.503383	1181.575	1187.713
		scale	0.00994643	0.0033083	0.0051826	0.019089		
	Gamma	shape	1.7561017	0.1952573	1.412233	2.1837001	1174.625	1180.762
		rate	0.0584203	0.0078796	0.0448493	0.0760978		
	Lognormal	meanlog	3.106759	0.0701543	2.96926	3.244259	1166.217	1172.355
		sdlog	0.854709	0.0531181	0.75669	0.965424		
	Gompertz	shape	0.00553358	0.0042831	-0.002861	0.0139283	1195.319	1201.457
		rate	0.02804405	0.003699	0.0216554	0.0363174		
	Log-logistic	shape	2.11457	0.153444	1.83423	2.43775	**1160.465**	**1166.603**
		scale	22.44123	1.479686	19.72067	25.53709		
	Generalized gamma	mu	3.157538	0.1079537	2.945952	3.369123	1167.844	1177.051
		sigma	0.842276	0.0564988	0.738511	0.96062		
		Q	0.129001	0.211207	-0.284957	0.542959		

Abbreviations: Sugema-Chemo, sugemalimab plus chemotherapy; Chemo, chemotherapy; AIC, akaike information criterion; BIC, bayesian information criterion.

Supplementary Table 2. Probability and Costs Associated with Adverse Events (Grade ≥3)

Adverse Event[a]	No. of patients (%)[b]	Costs in 2021 USD[c]	Reference	Disutility	Reference
**Sugemalimab plus chemotherapy**					
Anaemia	43 (13%)	3,667.90	(1)	0.072	(2)
Neutrophil count decreased	117 (37%)	3,183.70	(1)	0.348	(3)
White blood cell count decreased	45 (14%)	4714	(4)	0.072	(3)
Platelet count decreased	33 (11%)	6928	(5)	0.108	(6)
**Weighted average[c]**		**3063**		**0.159**	
**Chemotherapy**					
Anaemia	18 (12%)	3,667.90	(1)	0.072	(2)
Neutrophil count decreased	52 (33%)	3,183.70	(1)	0.348	(3)
White blood cell count decreased	27 (17%)	4714	(4)	0.072	(3)
Platelet count decreased	15 (10%)	6928	(5)	0.108	(6)
**Weighted average[c]**		**2984**		**0.147**	

[a]our analysis only included and evaluated grade ≥3 treatment-related adverse events.
[b]number within treatment arm: sugemalimab plus chemotherapy (N=320), chemotherapy (N=159).
[c]calculated as an average cost of toxicity using the weighted frequency of occurrence. This value was used in the base-case model.

References

1. Wu B, Dong B, Xu Y, Zhang Q, Shen J, Chen H, et al. Economic Evaluation of First-Line Treatments for Metastatic Renal Cell Carcinoma: A Cost-Effectiveness Analysis in a Health Resource-Limited Setting. *PLoS One* (2012) 7(3):e32530. doi: 10.1371/journal.pone.0032530
2. Freeman K, Connock M, Cummins E, Gurung T, Taylor-Phillips S, Court R, et al. Fluorouracil Plasma Monitoring: Systematic Review and Economic Evaluation of the My5-Fu Assay for Guiding Dose Adjustment in Patients Receiving Fluorouracil Chemotherapy by Continuous Infusion. *Health Technol Assess* (2015) 19(91):1-321, v-vi. doi: 10.3310/hta19910
3. Nafees B, Lloyd AJ, Dewilde S, Rajan N, Lorenzo M. Health State Utilities in Non-Small Cell Lung Cancer: An International Study. *Asia Pac J Clin Oncol* (2017) 13(5):e195-e203. doi: 10.1111/ajco.12477
4. Wong W, Yim YM, Kim A, Cloutier M, Gauthier-Loiselle M, Gagnon-Sanschagrin P, et al. Assessment of Costs Associated with Adverse Events in Patients with Cancer. *PLoS One* (2018) 13(4):e0196007. doi: 10.1371/journal.pone.0196007
5. Zheng H, Xie L, Zhan M, Wen F, Xu T, Li Q. Cost-Effectiveness Analysis of the Addition of Bevacizumab to Chemotherapy as Induction and Maintenance Therapy for Metastatic Non-Squamous Non-Small-Cell Lung Cancer. *Clin Transl Oncol* (2018) 20(3):286-93. doi: 10.1007/s12094-017-1715-1
6. Konidaris G, Paul E, Kuznik A, Keeping S, Chen CI, Sasane M, et al. Assessing the Value of Cemiplimab for Adults with Advanced Cutaneous Squamous Cell Carcinoma: A Cost-Effectiveness Analysis. *Value Health* (2021) 24(3):377-87. doi: 10.1016/j.jval.2020.09.014
